# On the Recent Differences of Opinion as to the Cause of Scurvy

**Published:** 1847-11

**Authors:** Andrew Anderson

**Affiliations:** The Surgeons to the Eye Infirmary, Glasgow


*On the Recent Differences of Opinion as to the Cause of Scurvy.* By Andrew Anderson, M. D., one of the Surgeons to the Eye Infirmary, Glasgow.—I have no intention of describing this disease. There has come under my notice but one fact in its history which I believe to be new: the occurrence of well-marked scorbutic amaurosis, and its cure by lemon juice. On this, however, I shall not at present linger, my object not being to fill up the history of scurvy, but to offer a very few remarks upon its cause.

Dr. Christison, in his interesting paper in the Edinburgh Monthly Journal (June, July, 1847,) proves that scurvy can, in certain cases, be cured by adding milk to the food, and concludes that fluid to be anti-scorbutic in virtue of the casein which it contains.

This opinion at once struck me as being at variance with that usually held; and, thinking that I could reconcile the two, I drew out the substance of the following remarks.

Since then, it has appeared that Drs. Lonsdale and Curran (Edinburgh Monthly Journal of Medical Science, and Dublin Quarterly Journal, for August, 1847) have, as I did, thought Dr. Christison partly in the wrong, and advanced proof that milk is not essentially, or at least not in all cases, anti-scorbutic. But as neither of these gentlemen has given that explanation of the matter which occurred to me, I venture to submit it still.

Dr. Christison's *observation* is, that in the Perth prison, scurvy broke out from treacle having been substituted for milk in the prisoners' diet, and that it disappeared when milk was given. He found that there was no deficiency in the mere quantity of solid food provided to them, and none in that of its nitrogenous elements; but that (excluding gluten, which has been proved to be, when taken alone at least, not nutritious) there was in the food a marked deficiency of *nutritive azotised matters*,—viz. fibrin, albumen, and casein.

His *deduction* is, that it was the want of these which caused the scurvy; and that it was by supplying such matter, in the shape of casein, that milk proved anti-scorbutic.

Now in the first place it must be obvious to all, that this doctrine will not account for every outbreak of scurvy, or explain every cure; for it is notorious that lemon juice, without a drop of milk, or any nitrogen, has prevented and cured the disease in instances quite innumerable. But the question is, may not scurvy be the offspring of different causes, and owe its origin now to the absence of fresh vegetables, and again to that of nutritious azotised food?

It seems to me that this must be answered in the negative; and that it is not very difficult to reconcile the opposite opinions, and to show how lemon juice and milk may both, and both in one way, be anti-scorbutic.

Briefly to recall facts, we know—

1. That it has been proved by authors, that while any debilitating and depressing agency may predispose to scurvy, its essential cause is improper food.

2. That food, to support human life, must contain fibrin, albumen, or casein—it signifies not whether they be of animal or of vegetable origin: and that to provide sufficient fuel for maintaining animal heat, non-azotised substances must in general be added, no matter whether they be amylaceous, saccharine, or oily.

3. That if nitrogenous food be absent, the body cannot be nourished, give as much of the other as you choose; and that if the latter be withheld, and the former taken in quantity sufficient to supply carbon to the oxygen absorbed, the system is apt to get loaded with superfluous nitrogenous matter, and gout, or worse, may follow.

But how is it that, both being given, the blood, if they alone be given, still becomes depraved, and scurvy is the consequence? It were easy to see a reason for this, if in all instances in which scurvy occurs, the quantity of nourishing food were, as in the Perth prison, too small; but there is abundant evidence to disprove this. Thus Dr. Curran (loc. cit.) records a very bad case, in the person of a woman who had been fed on plenty of butcher meat, and bread and butter, tea and coffee, wine and porter. Dr. Ritchie saw one parallel to this; and during the discussion on Dr. Christison's paper in the Edinburgh Medico-Chirurgical Society (Edinburgh Journal of Medical Science, June,) it was stated that the disease had occurred in a boy overfed with animal food, and in a lady whose diet was butcher's meat and milk. In these instances the food abounded in both varieties of the elements needful for nutrition, and yet it did not nourish well. How was this?

To find what was yet required, let us revert to the history of the prevention and cure of scurvy by lemon juice. This substance contains but 2 per cent. of solid matter, of which 1.77 is citric acid.—(Christison's Dispensatory, *in loc.*) Four ounces daily will shortly cure a bad case of scurvy, and yet can contain no more than forty grains of solids—solids, too, which are not nitrogenous. This cannot possibly furnish pabulum to make the blood nutritious; and yet it removes the malady. The lady whose case I have just cited was rapidly cured by oranges, her diet being otherwise unchanged; in explanation of which I venture to suggest the following hypothesis:—

Food appropriate to man must consist of three parts—the nitrogenous, to nourish; the non-nitrogenous, to produce the extra heat required; and a third element, to aid in the assimilation of these. This element is furnished by the various, usually more or less acid, juices contained in vegetables and fruits. In favourable circumstances man may dispense with these, and live in tolerable health, but withal obnoxious to disease; and if he be weakened, as by confinement, or depression, or previous disease; or if the nourishment in the food be moreover scanty, as it was in the case of the prisoners at Perth, scurvy comes on.

All the substances eminent as anti-scorbutics contain more or less acid; and it is proved that the more acid the fruit, as Dr. Trotter found of gauvas, the greater is its virtue.

I presume then that these juices and acids act, not by themselves supplying nourishment for the blood, but by in some way promoting the assimilation of the nutritive part of the food: and I would compare their efficacy in scurvy to that of iron in anæmia. The anæmic patient may be fed exceedingly, but he cannot assimilate the nutriment he digests without the aid of a metal which itself can be no pabulum to the blood. And so the scorbutic may eat enormously of flesh, which is blood, (Liebig,) yet cannot change it into *his own* blood without the aid of certain vegetable matter; an aid which seems essential, though how it is given no man can tell. Vinegar and nitre, and one or two substances besides, have been found to cure scurvy now and then, (by Henderson, &c.,) but they have failed at other times. They probably exert in the process of assimilation an agency more or less like that of citric acid, although not so uniform or powerful; and so I suppose that milk may, by means of the *lactic acid* produced while it is digested, and not by means of its casein, effect in some instances the cure of scurvy. If it did so in virtue of the casein it contained, cheese should have the same effect,—a thing I never heard of. And milk, like vinegar and nitre, seems to fail more often than it prospers; for Drs. Curran, Lonsdale, and Ritchie record cases of the disease as having happened in persons making free use of milk.

When the diet is nitrogenous enough, lemon juice will be more useful; but when, as in the Perth prison, it is not so, milk may be the best anti-scorbutic, because it will supply the essential food of the blood, as well as what I may call its condiment. That is, milk alone may in such a case be preferable to lemon juice alone; but, no doubt, "both are best."

Why is there sugar in milk? Not to form flesh of course. Not to produce caloric either; butter would serve that purpose well enough. The casein to nourish,—the oil to heat; is it not possible that the lactine may be intended to promote the assimilation of these by being first changed into lactic acid, (as all nurses know it is,) and by then supplying to the infant the want of that vegetable food which he does not receive, and could not digest; for the purpose, in short, of preventing scurvy? Dr. Andrew Buchanan well suggests that the *experimentum crusis* were to try whether *whey* would cure a man of scurvy.

And how do potatoes nourish men so well (*teste* Hibernia) that no other article of food will singly keep them in such health? Mr. Crum has shown (Philosophical Society of Glasgow) that four pounds of potatoes contain about as much albuminous matter as an egg:—the root then affords starch to heat, and nitrogen to nourish; but peas and beans, which do as much, will not serve as man's sole food. They are deficient in the third, the anti-scorbutic element, which Baly has shown to be possessed by the potatoe ; hence its surpassing value,—it is *milk for men.*

May we not in some such way as this reconcile Dr. Christison's observations with common notions ? The analyses which I am about to quote prove that there is no necessary lack of fibrin in scorbutic blood ; nay, that even in severe cases that element may be unnaturally plentiful, and the corpuscles, moreover, not much diminished. The darkness of the colour of the clot, and its occasional jelly-like consistency, indicate no doubt a depraved condition of the blood : but the startling rapidity of the cure by lemon-juice appears to show that the error is in its vital structure, rather than in its chemical composition ; and is repaired by putting into their right places, as it were, nutritive molecules already present in the fluid, rather than by the more tedious process of absorbing and adopting new materials.

When scurvy, however, does break out, it soon brings on anæmia; and then, of course, the corpuscles and the albumen are both progressively reduced in quantity, while the fibrin and salts remain unchanged. This will be seen in the last analysis which I shall quote which yet must not lead us to confound the essential with the accidental.

But Dr. Curran, too, has a chemical theory of scurvy :—

"Dr. Christison, by overlooking *the salts* contained in food, has committed an error which totally destroys all the value of his reasonings. That vulgar experience, which leads the bird-fancier to supply his birds with lime, should not be lost upon those who have to direct the nutrition of the masses of the human species. Dr. Aldridge is, however, the only chemist who has made his knowledge practically available on this subject; and from his excellent little work, I extract the following short, intelligible, general directions." (*Loc. cit.*. pp. 113–14.)

Dr. Aldridge's theory, it would appear, is, that it is the want of lime and saline matter in the food which causes scurvy. This were remarkable, if true,—because if so, how should pure citric acid cure the malady ; and why, in the celebrated historical case of the continental army, did dry herbs, containing of course all their salts, fail to alleviate the scurvy like the fresh plants. But we need not discuss this point ; for Dr. Aldridge's theory is not more correct than new. It is simply a *rechauffée* of that which, broached by Dr. Stevens fifteen years ago, was refuted by Dr. Kerr in the Cyclopædia of Practical Medicine (iii. 694.) But as it was possible that Dr. Aldridge might have analysed scorbutic blood before constructing his little work, I searched for his experiments, but in vain. Yet the materials may be found from which to form our judgment : in a paper by M. Fauvel, in the *Archives Générales* (Août 1847,) there are recorded some analyses of such blood by the experienced hands of MM. Becquerel and Rodier: and these I copy, as well as a portion of two imperfect ones from Dr. Ritchie's paper.

| B. and R.'s Analysis of (See Simon's Animal Chemistry, i. 233, 234,) FEMALE BLOOD, HEALTHY. | Average of two of Dr. Ritchie's cases. | Average of two cases of little severity. | Average of two cases of much severity. | A very severe case indeed. |
|---|---|---|---|---|
| | Average. | | | | |
| Corpuscles, | 127. | | 116. | 110. | 79. |
| Fibrin, | 2·2 | | 2.8 | 3·8 | 2.2 |
| Animal matter in serum, | 72. | | 65. | 67. | 56. |
| Saline matter in serum, | *6·8 | *6· | 6·4 | 6·5 | 7·8 (Anæmia coming on.) |

*I have deducted 0.54 in this analysis for the iron, as not forming part of the saline matter of the serum.

Hence we perceive that in point of *fact* the worst scorbutic blood may contain rather *more* saline matter than the healthy fluid does. May I venture to add, that

Dr. Curran, by overlooking the salts contained in the *blood*, has committed an error which totally destroys all the value of his reasonings. That vulgar prudence which leads the reasoner to base his theories on facts, should not be lost upon those who wish to direct the opinions of the members of the medical profession. MM. Bacquerel and Rodier are, however, the only chemists who have acquired any available experimental knowledge on this subject; and from their excellent little production I have extracted the preceding short, very intelligible, and particular analysis.

That scurvy is a "blood disease" no man can doubt; and I suspect that British blood has, by the past year of dry feeding, been depraved more generally than may be thought. All the blood drawn by my directions, during the last three months, from patients of the Glasgow Eye Infirmary (who presented no external signs of scurvy,) has been of most unhealthy aspect: the clot large, soft and dark, like black currant jelly; and the buffy coat, when present, gelatinous and pale.

It also appears to me indubitable, that scorbutic blood may be found in many differently perverted states. My friend Dr. Ritchie (Edinburgh Monthly Journal, August, p. 83,) has met with some containing just half the usual quantity of fibrin; in other cases we have seen that substance, on the contrary, augmented. The scorbutic state may be complicated with various other conditions of the system, which will of course tend to produce changes on the vital fluid, just as pyrexial blood varies according to the local lesion present. The effect of treatment furnishes a proof of this; for while bleeding is usually most hurtful in scurvy, and mercury quite a poison, cases do occur in which each of these means is indeed remedial. (Dr. Bogie in Lonsdale, *loc. cit.* p. 102.)

It is reasonable also to conclude, that when anæmia has come on, the treatment peculiarly suited to that state may with advantage be combined with that for scurvy; so that while the simple examples of the disease may, as I have seen in patients of my own, be cured by pure citric or tartaric acid, the anæmic forms, in which the lips and the exuding blood are pale, will, as in a lady about whom I was consulted, be, by the use of iron, hurried on more rapidly to health.—*Monthly Journal of Medical Science.*

